# Effects on patients of their healthcare practitioner's or institution's participation in clinical trials: a systematic review

**DOI:** 10.1186/1745-6215-12-16

**Published:** 2011-01-20

**Authors:** Mike Clarke, Kirsty Loudon

**Affiliations:** 1 UK Cochrane Centre, National Institute for Health Research, Middle Way, Oxford, OX2 7LG, UK; 2 School of Nursing and Midwifery, Trinity College, Dublin 2, Ireland

## Abstract

**Background:**

Systematic reviews have shown uncertainty about the size or direction of any 'trial effect' for patients in trials compared to those treated outside trials. We are not aware of any systematic review of whether there is a 'trial effect' related to being treated by healthcare practitioners or institutions that take part in research.

**Methods:**

We searched the Cochrane Methodology Register and MEDLINE (most recently in January 2009) for studies in which patients were allocated to treatment in one or other setting, and cohort studies reporting the outcomes of patients from different settings. We independently assessed study quality, including the control of bias in the generation of the comparison groups, and extracted data.

**Results:**

We retrieved and checked more than 15,000 records. Thirteen articles were eligible: five practitioner studies and eight institution studies. Meta-analyses were not possible because of heterogeneity. Two practitioner studies were judged to be 'controlled' or better. A Canadian study among nurses found that use of research evidence was higher for those who took part in research working groups and a Danish study on general practitioners found that trial doctors were more likely to prescribe in accordance with research evidence and guidelines. Five institution studies were 'controlled' but provided mixed results. A study of North American patients at hospitals that had taken part in trials for myocardial infarction found no statistically significant difference in treatment for patients in trial and non-trial hospitals. A Canadian study of myocardial infarction patients found that trial participants had better survival than patients in the same hospitals who were not in trials or those in non-trial hospitals. A study of general practices in Denmark did not detect differences in guideline adherence between trial and non-trial practices but found that trial practices were more likely to prescribe the trial sponsor's drugs. The other two 'controlled' studies of institutions found lower mortality in trial than non-trial hospitals.

**Conclusions:**

The available findings from existing research suggest that there might be a 'trial effect' of better outcomes, greater adherence to guidelines and more use of evidence by practitioners and institutions that take part in trials. However, the consequences for patient health are uncertain and the most robust conclusion may be that there is no apparent evidence that patients treated by practitioners or in institutions that take part in trials do worse than those treated elsewhere.

## Background

The involvement of increasing numbers of patients in clinical research, in particular randomised trials, is necessary to resolve uncertainties about healthcare interventions. A Cochrane methodology review has brought together evidence comparing the outcomes of patients treated in randomised trials with similar patients who received similar treatments outside trials [1]. It found continuing uncertainty about the size or direction of any 'trial effect' for patients in trials but, in general, participation in trials was not associated with any obvious benefit or harm.

We report here a systematic review of whether there is a trial effect at the level of healthcare practitioners or institutions. We set out to answer the question: do patients who are treated by practitioners or in institutions that take part in trials have different outcomes to patients treated elsewhere? The answer to this question is important to patients who might wish to use the evidence when making a choice about where they receive their health care and from whom, or who wish to use it as a guide to the quality of care they will receive. It is also important that policy makers and others involved in decisions about practitioners and institutions have access to evidence on possible relationships between the participation in research of practitioners or institutions and the outcomes of their non-trial patients. We examined this with research that has looked at health outcomes for patients, the uptake of evidence from research, and adherence to practice guidelines. A systematic review is necessary to avoid undue emphasis on the findings of individual studies, which may have found beneficial or harmful effects. This review was commissioned by the National Institute for Health Research, including an assessment of the effects on patients of their own participation in clinical trials, supplementing the work of Vist et al [1], but that is not reported here as it confirmed their findings.

We sought to assess patient related outcomes in the following situations: (1) patients treated by healthcare practitioners who take part in clinical trials versus similar practitioners who do not take part; and (2) patients treated in institutions that take part in clinical trials versus those treated in similar institutions that do not take part in such trials. In doing this, we recognised that there is a possibility of clustering due to the association between the practitioner and the institution (i.e. would any effect detected be due to the practitioner or to the institution within which they work?). We tried to compensate for this by placing most emphasis on studies in which there was a mixture of research and non-research practitioners in an institution. We also sought to distinguish between the outcomes for patients being treated in trials, with those being treated outside of trials by the research practitioners or institutions; and to assess the extent to which the studies we identified dealt with the confounding that might arise if the patients being treated by research practitioners or in research institutions are fundamentally different to the patients in non-researching settings.

## Methods

We conducted the review in accordance with the methods used within The Cochrane Collaboration for systematic reviews of methodology [2].

### Eligibility criteria of studies

#### Types of study

Studies that reported empirical comparisons of the different situations were eligible if patients were randomised or allocated by other means to treatment by research versus non-research practitioners, or to treatment in a research versus a non-research institution., Cohort studies in which the outcomes of patients treated by research versus non-research practitioners, or in a research versus a non-research institution, or both were also eligible. We did not generate our own comparisons between groups of patients through, for example, comparing independent reports of patients treated in institutions that took part in trials and other reports of patients treated in non-trial institutions. All types of institution providing health care, all types of healthcare practitioner, and all types of patient (including people who were healthy and, for example receiving interventions to prevent illness) were eligible.

#### Types of outcome measure

The primary outcome measure was the health of patients, as reported in each included study. In recognition of the fact that such outcomes may be poorly reported and may be subject to confounding that is difficult to correct for (for example, because of variations in the referral patterns for hospitals that take part in research and those that do not), secondary outcomes included the uptake of the findings of research and adherence to practice guidelines.

### Search methods for identification of studies

Searching for studies of relevance to reviews similar to this has been noted to be especially difficult, not least because of the absence of suitable search terms within medical literature databases [3,4]. Therefore, we began our searches with the Cochrane Methodology Register, which has been compiled as a resource for articles relevant to the methodology of evaluations of health and social care [5]. It contained more than 11,000 bibliographic records, as of January 2009. We searched the version of the Register from Issue 1, 2009 of *The Cochrane Library*. Records in the Register are assigned index terms from a taxonomy that is maintained by the Cochrane Methodology Review Group. We retrieved all records with the following index terms: 'Applicability and recommendations - Assessments of the impact of research', 'Applicability and recommendations - Levels of evidence and strength of recommendations', 'Applicability and recommendations - Recommendations', 'Consumer involvement', 'Eligible and randomised versus eligible and not randomised' and 'Patient involvement'.

Two researchers (TC and MC) checked each record independently to identify reports that might be eligible for the review. The records identified as eligible or potentially eligible from the Cochrane Methodology Register were used as the 'seeds' for related articles searches in PubMed. These searches were done in December 2008. The records for 3964 related articles were checked independently by two researchers (TC and MC). Finally, a specially designed search strategy was prepared for MEDLINE (Additional File 1). This search was run on 23 January 2009 using OvidSP MEDLINE, for the period from 1950 to mid-January 2009, with no language or publication year restrictions. All 9820 retrieved records were checked independently by two researchers (TC and MC). The references in the reports of included studies were also checked for relevance by KL and MC. Full text copies of potentially eligible articles were obtained, and checked by one of two researchers (KL or MC), who then discussed and agreed on the eligibility of each study. Although searches of other databases or sources might have revealed further studies, the restriction to the Cochrane Methodology Register and MEDLINE was influenced by the comprehensive nature of the processes that have been used to compile the former and the resources available for this review.

### Data extraction and analyses

The following information was extracted for each study: reference; population and setting or interventions studied (including details on the practitioners and institutions, if appropriate); means used to establish the trial group and the comparator group; confounders and how these were adjusted for (including different diagnoses of patients or different treatments); outcomes (including patient health, use of evidence from trials or guidelines); and a summary of the relevant results. These data were extracted by one researcher (KL) and checked by a second (MC).

The risk of bias in each study was assessed by considering the control of selection bias in the groups being compared. We judged a study to be 'well controlled' if it used random allocation to allocate patients to be treated by practitioners who do take part in trials versus practitioners who do not take part in trials, or in institutions that do take part in trials versus institutions that do not take part. Studies were categorised as 'controlled' if random allocation was not used but attempts were made to control for all confounding factors or if it was reported that the study contained no imbalances in confounding factors. Studies that reported imbalances in confounding factors and did not attempt to control for these in the analyses were categorised as 'poorly controlled'. These categories are similar to those used in the Cochrane methodology review investigating the possibility of a trial effect in patients in research studies [1].

## Results

### Overall

The searches identified a total of 15,149 records. Following our independent screening of these and the checking of reference lists, we identified 21 potentially eligible studies for practitioners, 17 for institutions, and 10 that were initially unclassified. Full copies of the articles were obtained and checked against the eligibility criteria, leading to a total of 13 articles for this review. There were 5 articles for the review of practitioners and 8 articles for the review of institutions. The included studies are summarised in Table 1. We were unable to do a meaningful meta-analysis within any of the categories because of heterogeneity across the individual studies, and the lack of a significant difference in a study should not be taken as evidence that there truly is no difference since the studies were not necessarily adequately powered to detect a difference should there be one, in either direction.

**Table 1:** Summary of included studies

Reference	Type of participants	Intervention	Outcome measures	Brief summary
**Practitioners**				

**6**	General Practitioners in Denmark	Treatment for gastro-oesophageal reflux disease	Drug prescription for individual patients	GPs who took part in trial significantly more likely to prescribe 'trial drug' proton pump inhibitors than other GPs.

**7**	Nurses in Canadian teaching hospital	Research working groups	Nurses attitudes to research, access to research, support of the use of research, use of research	Post intervention scores were higher for nurses involved in high or low research participation units than for nurses in the units that did not take part in these groups.

**8**	Gastrointestinal specialists	Management of Barrett's oesophagus	Adherence to international guidelines, especially in regard to tissue sampling	Adherence to guidelines increased after the trial started, particularly in trial centres.

**8**	Hospital doctors	Treatment for myocardial infarction	Medication received on discharge compared to admission	Doctors involved in trials change prescribing practice quicker than physicians in routine practice.

**10**	Surgeons in 3 adjacent counties in England	Discharge decisions following inguinal hernia and varicose veins surgery	Mean length of hospital stay	The mean length of stay decreased significantly in the trial area, with less effect in control areas.

**Institutions**				

**11**	Trial (high and low participation) and non-trial hospitals in CRUSADE (Can Rapid Stratification of Unstable Angina Patients Suppress Outcomes With Early Implementation of the American College of Cardiology/American Heart Association Guidelines), North America	Treatment for patients with high-risk non-ST-segment elevation acute coronary syndrome with unstable angina and non-ST segment elevation acute coronary syndrome	All cause mortality and adherence to guidelines	In-hospital mortality was lower in high trial participation hospitals compared to low participation and non-trial hospitals. Greater adherence to guidelines in high trial participation hospitals compared to low participation and non-trial hospitals.

**12**	Acute care hospitals in Ontario, Canada participating in trials and those not.	Treatment for myocardial infarction	Hospital mortality and treatment information	Patients in trial hospitals fared better than patients in non-trial hospitals

**13**	Gynaecological departments in hospitals in Germany taking part in clinical trials and hospitals not taking part	Treatment for ovarian cancer	Survival from diagnosis to date of last follow up or death for up to two years after diagnosis and adherence to treatment guidelines.	Treatment in hospitals that did not participate in trials was associated with significant risk of death (adjusted for differences in stage of patients)

**14**	General practitioners in trial and non-trial practices in Denmark	Management of asthma	Physicians' adherence to international treatment recommendations and use of trial sponsors drugs	No change in adherence to guidelines in trial practices but there was an increase in prescription of sponsor drugs in trial practices

**15**	Hospitals in North America involved in the Survival and Ventricular Enlargement trial (SAVE) and in the Multicenter Diltiazem Postinfarction Trial (MDPIT) and hospitals not involved in these trials.	Treatment for myocardial infarction	Prescribing of angiotensin-converting inhibitors (ACE) at hospital discharge	No significant differences between trial and non-trial hospitals.

**16**	Cancer care facilities: community hospital, community cancer centre and teaching/research facilities in the USA	Treatment of patients with advanced stage laryngeal cancer	Chemotherapy and radiation treatment to patients	Higher percentage of patients with advanced-stage laryngeal cancer were treated with chemo-radiation in teaching/research hospitals than in community hospitals and community cancer centres

**17**	Apheresis units in major medical centres in Canada	Treatment of multiple sclerosis, thrombotic thrombocytopenic purpura, and myeloma cast nephropathy	Apheresis	Large increase in apheresis use, particularly outside trials, labelled 'jumping the gun' by the research team.

**18**	Centres taking part in ADEBAR trial of chemotherapy for women with breast cancer	Treatment for breast cancer	Changes to treatment strategies and patient care since participating in ADEBAR	Prior to ADEBAR, 63.2% of centres had not entered high-risk breast cancer patients into a clinical trial and 44.2% of patients received inadequate treatment by current standards.

### Practitioners

We identified five studies comparing patient-care related outcomes for practitioners who took part in clinical research versus outcomes for practitioners who had not taken part in clinical research [6-10]. The studies were heterogeneous and their findings are presented individually, with no meta-analyses. Two trials were judged to be 'controlled' or better [6,7] and these are described first in this section.

Meineche-Schmidt et al reported a study called ONETWO which investigated the impact of taking part in a randomised trial (ONE) of 'on demand' versus treatment courses for the management of gastro-oesophageal reflux disease. They compared prescribing patterns among 64 general practitioners (GPs) who took part versus a random sample of 58 other Danish GPs [6]. The study compared the outcomes of 247 patients treated by the GPs within ONE, 451 similar patients treated by the same GPs outside ONE, and 469 similar patients treated by the comparator GPs outside of ONE. GPs who took part in the trial were significantly more likely to prescribe 'on demand' proton-pump inhibitors to their patients (322 of 698, 47%) than was the case for other GPs (129 of 469, 27%) (P < 0.0001). We judged this study to be 'controlled' because there were few baseline imbalances between the trial and non-trial GPs (on sex, geography, number of years as doctors and distribution by region or type of practice) or between their patients (on age and sex, but symptoms were significantly lower at baseline in the trial patients), and the means for establishing the control group was unlikely to introduce major bias.

Tranmer et al conducted a randomised trial in a teaching hospital in Canada in which nurses in two medical surgical units were allocated to a high level of participation in research, nurses in two other units were randomised to a low level of participation and nurses in the final two units were randomised to no participation [7]. After randomisation, nurses in the high- and low-participation units were invited to join the relevant research working groups. Eighteen and ten nurses, respectively, volunteered. Information on the use of research was sought through a self-reported questionnaire before the intervention and one year after from a total sample of 190 nurses. It was found to increase in all units, with no significant differences between the three types of unit. The post intervention scores were higher for nurses who took part in the research working groups in the high- and low-participation units than the nurses that did not take part in these groups. We judged this study to be 'well controlled' because of the use of randomisation to create the different levels of exposure to research. However, the sample size was small and cluster randomisation was used, with only two medical surgical units in each of the three interventions.

The three other studies were judged to be 'poorly controlled' because of the possibility of differences between the patients seen by trial and non-trial practitioners [8], or because health outcomes between these two groups of patients might have been influenced by the use of different treatments for them [9], or both [10].

Das et al compared practice in the UK centre that recruited most patients to a randomised chemoprevention trial, the Aspirin Esomeprazole Chemoprevention Trial (AspECT), before and after the start of the trial [8]. They audited biopsies to compare AspECT patients with non-AspECT patients in these two periods. They found no statistically significant difference between AspECT and non-AspECT groups while AspECT was ongoing. However, both these patient groups had significantly more biopsies per cm length of Barrett's oesophagus compared to similar patients treated before AspECT. We judged this study to be 'poorly controlled' because the report does not mention (or appear to control for) changes over time, which might have influenced the change in the number of biopsies independently of involvement in the randomised trial. Such changes might have occurred because of, for example, changes in other treatments or in routine practice.

Kizer et al investigated the uptake of the findings of randomised trials and recommendations from guidelines during 1978 to 1995 for patients needing treatment for the secondary prevention of myocardial infarction who were enrolled in the Multicenter Investigation on Limitation of Infarct Size (MILIS) and Thrombolysis in Myocardial Infarction (TIMI) trials [9]. They compared medication use by non-trial and trial physicians using data on medication use at enrolment as the guide to decision making by non-trial physicians and data at discharge as the guide to decision making by trial practitioners. Effective drugs (aspirin, beta-blockers, angiotensin-converting enzyme (ACE) inhibitors) tended to be more common at discharge than at enrolment. The reverse was true for an ineffective class of drugs (calcium channel blockers). The authors concluded that they had demonstrated 'prompt responses by physicians who design or implement randomized clinical trials to the results of RCTs and study overviews' which they contrast with 'enduring deficiencies in the application of RCT results by physicians in routine practice, despite publication of synthesizing overviews and task force guidelines.' We judged this study to be 'poorly controlled' because the report does not adjust for differences over time in patient characteristics which might have influenced the prescription or use of the medications investigated.

Adler examined the discharge decisions of surgeons in three adjacent areas of England. A randomised trial had taken place in one of these areas [10]. The comparison was made for 1970 and 1975, studying the impact of the 1973 release of the preliminary results of the randomised trial, which favoured early discharge following inguinal hernia and varicose veins surgery. Large changes were seen in the study area but not in the adjacent, control health districts. The mean length of stay decreased significantly in the trial area and there was less effect in the control areas. The author concluded 'the research findings played a part in changing clinical practice in the study area'. We judged the study to be 'poorly controlled' because no information was presented on why the control areas were chosen (in preference to other areas adjacent to the area of the trial) and the analyses did not adjust for potential confounders, such as the types of patient in the different areas or the types of treatment provided in the different areas.

### Institutions

We identified seven studies (four of which we judged to be 'controlled') which compared patient-care related outcomes for institutions that had taken part in clinical research versus outcomes for institutions that had not taken part in clinical research [11-17]. and one study which compared the uptake of a trial's results by institutions that had and had not taken part in it [11]. We were unable to do a meaningful meta-analysis because of heterogeneity across the individual studies and therefore present the results of each study separately, beginning with the five studies judged to be 'controlled' [11-15]. The first of these is the study of the uptake of a trial's results by participating and non-participating hospitals.

Majumdar et al investigated the impact on practice of being part of a myocardial infarction trial that showed a treatment (ACE inhibitors) to be beneficial and of another trial that showed a treatment (calcium channel blockers) to be ineffective; in hospitals that were taking part in a third randomised trial, GUSTO-1, in North America [11]. They compared the 22 hospitals that took part in the Survival and Ventricular Enlargement (SAVE) trial from 1987 to 1990 with hospitals that did not take part in it; and the nine hospitals that took part in the Multicenter Diltiazem Post-Infarction Trial (MDPIT) with those that did not. There was no statistically significant difference in the percentage of patients who received an ACE inhibitor at discharge in hospitals that had taken part in SAVE compared to non-SAVE hospitals. The adjusted odds ratio was 1.1 (95% CI 0.8 to 1.4, P = 0.67). There was no statistically significant difference in the percentage of patients who received a calcium channel blocker at discharge in hospitals that had taken part in MDPIT compared to non-MDPIT hospitals (adjusted OR 0.8, 95% CI 0.5 to 1.3, P = 0.58). We judged this study to be 'controlled' because potential confounders were adjusted for in the analyses and the analyses also accounted for statistical clustering of patients treated at the same hospital.

From 1989 to 1993, Jha et al studied five groups of acute myocardial infarction patients in Ontario, Canada using data on hospital admissions [12]. Four of these groups were participants and non-participants in hospitals taking part in each of two thrombolysis studies: GUSTO (Global Utilization of Streptokinase and Tissue Plasminogen Activator for Occluded Coronary Arteries) and LATE (Late Assessment of Thrombolytic Efficacy). The fifth group consisted of patients from other hospitals not participating in these trials. There were 30 GUSTO hospitals, 10 LATE hospitals and 165 external hospitals. Hospital mortality was higher at the non-trial hospitals (17.4%) than it was for trial participants (GUSTO: 6.9%; LATE: 6.6%) at the trial hospitals but similar to that for non-participants in those hospitals (GUSTO: 16.8%; LATE: 19.7%). After adjustment for patient characteristics, survival in the trial hospitals was higher among participants than non-participants (GUSTO: odds ratio 1.8, 95% CI 1.4 to 2.2; LATE: OR 2.1, 95% CI 1.2 to 3.6), and it was higher among trial participants than non-trial patients in external hospitals (GUSTO: OR 1.8, 95% CI 1.5 to 2.4; LATE: OR 1.8, 95% CI 1.0 to 3.2). However, survival for non-trial patients at GUSTO hospitals was similar to that for patients at external hospitals (OR 1.0 95% CI 0.9 to 1.1), and it was lower for non-trial patients at LATE hospitals (OR 0.8 95% CI 0.8 to 1.0) compared to patients at external hospitals. We judged this study to be 'controlled' because analyses were adjusted for potential confounders.

A study of gynaecological departments in 165 German hospitals sought data on the treatment and two year survival of ovarian cancer patients diagnosed in the third quarter of 2001 [13]. Eighty of these hospitals were involved in clinical trials conducted by one of two co-operative groups in Germany: the Ovarian Cancer Study Group and the Northeastern Society of Gynaecologic Oncology, and 85 hospitals were not participating in these trials. Treatment in a non-trial hospital was associated with a significantly increased risk of death (hazard ratio 1.82, 95% CI 1.27 to 2.61, P = 0.001) after adjustment for baseline factors. Measures of adherence to treatment guidelines also favoured patients treated in study hospitals. We judged this study to be 'controlled' because analyses were adjusted for potential confounders.

Andersen et al investigated adherence to asthma guidelines and use of drugs manufactured by the trial's sponsor, in relation to the participation of general practices in a trial [14]. Ten practices taking part in the trial (SymbiAC) of an asthma treatment were compared with 165 non-trial control practices in Denmark in the early 2000s. There was no significant effect of trial participation on guideline adherence comparing the second year after participation in the trial with the year before participation (odds ratio for the second year for trial versus non trial practices: 1.00, 95% CI 0.84 to 1.19). Prescribing of the trial sponsor's drug rose from 17.4% to 55.4% in trial practices, and from 14.8% to 40.5% in non-trial practices (not significantly different between trial and non-trial practices). However, the sponsoring company's share of the total prescribing volume of asthma drugs increased significantly more in trial practices than in non-trial practices (OR 1.26, 95% CI 1.04 to 1.54). The authors concluded that their study was not able to detect reliably any impact on guideline adherence but that their study 'confirms the hypothesis that physician involvement in clinical trials is a powerful tool for influencing company-specific drug preferences'. We judged this study to be 'controlled' because the analyses adjusted for baseline imbalances between the practices. Furthermore, the choice of control practices is unlikely to introduce major bias, because it included almost all of the practices in the same region as the trial practices.

For the period 2001 to 2006, Majumdar et al compared US hospitals that had no trial participation (145 hospitals), low trial participation (226 hospitals, median 1% of patients enrolled in trials) and high trial participation (123 hospitals, median 4.9% of patients enrolled in trials) on hospital mortality and adherence to guidelines for 174,062 patients with high-risk non-ST-segment elevation acute coronary syndrome [15]. Patients treated at hospitals that participated in trials had significantly lower mortality than patients treated at non-participating hospitals: 5.9% for non-trial hospitals, 4.4% for low-participation hospitals and 3.5% for high-participation hospitals (adjusted P-value for trend 0.003). The comparison of low-participation hospitals versus non-trial hospitals produced an adjusted odds ratio of 0.9 (95% CI 0.8 to 1.0, P = 0.04) and the comparison of high-participation hospitals versus non-trial hospitals produced an adjusted odds ratio of 0.8 (95% CI 0.7 to 0.9, P = 0.003). A composite guideline adherence score was also used to assess the uptake of nine recommendations from guidelines. This increased with increasing trial participation: 76.9% versus 78.3% versus 81.1% for non-trial, low-participation and high-participation hospitals, respectively (adjusted P-value for trend 0.008). We judged this study to be 'controlled' because analyses were adjusted for potential confounders. This was done for hospital and patient characteristics for the hospital mortality analyses and for hospital characteristics for the analyses of guidelines adherence.

The three other studies of the possibility of a trial effect for institutions were judged to be 'poorly controlled' because of the possibility of differences between the patients in the different institutions [16-18].

Three types of American institutions involved in the care of patients with cancer were studied from 1985 to 2001 by Chen et al: community hospitals, community cancer centres and teaching/research facilities [16]. The impact of the 1991 publication of a randomised trial demonstrating similar survival between non-surgical therapy (chemo-radiotherapy) and total laryngectomy for patients with advanced laryngeal cancer was investigated. Over the whole period, patients in teaching/research facilities were more likely to be treated with chemo-radiotherapy (14.2%), compared with community hospitals (12.9%) and community cancer centres (13.1%). The percentage of patients with advanced laryngeal cancer treated with chemo-radiotherapy increased overtime in all centres but use of chemo-radiotherapy increased at a significantly faster rate in community cancer centres and teaching/research facilities after the publication of the VA Laryngeal Cancer Study. We judged this study to be 'poorly controlled' because differences in the demographic and clinical characteristics of the patients at the different types of institution do not appear to have been accounted for in the analyses.

Clark et al studied data on apheresis use in 19 units taking part in a randomised trial of apheresis for myeloma cast nephropathy and five non-trial units (1998 to 2000) [17]. They found increased use of apheresis in both types of institution. In trial centres, the number of patients undergoing apheresis increased from 62 to 127 per year (with 35 of the 127 being patients in the trial). In non-trial centres, the annual number increased from 71 to 88. We judged this study to be 'poorly controlled' because there was no discussion of potential confounding variables between the trial and non-trial centres, such as those relating to the case mix of patients which might have arisen due to different referral patterns.

Centres taking part in the German ADEBAR randomised trial of chemotherapy regimens for women with high-risk breast cancer were studied by Janni et al, before and after they recruited patients to the trial [18]. The study period ran from 2001 to 2004, and 98 (51%) centres responded to the questionnaire with 95 of these providing data that could be used in the analyses. Before their participation in ADEBAR, 63% of the centres had not entered high-risk patients into a clinical trial and 44% of patients treated before ADEBAR were judged to have received inadequate treatment by current standards. Following their participation in ADEBAR, 80% of centres reported an improvement in professional knowledge relevant to breast cancer and 31% of centres found that patient care improved. We judged this study to be 'poorly controlled' because it contains insufficient information on the control of confounders (such as patient characteristics), the response rate to the questionnaire was low and it relies on self-reported data.

## Discussion

Taken together, the studies of practitioners and institutions suggest that there might be a 'trial effect' of better outcomes, greater adherence to treatment guidelines and more use of evidence-based practice in settings that take part in trials compared to non-trial settings, but these findings should be treated with caution and require examination in further, well designed studies (see below). There is a lack of consistency in the findings across the studies, with some reporting a statistically significant trial effects and others reporting no significant differences between research and non-research settings. This may be due to a lack of an effect or the lack of sufficient power to detect a true effect, but the considerable heterogeneity we encountered means that it was not possible to assess this through meta-analyses.

If there is a trial effect, its magnitude is uncertain and it is possible that the findings are due to bias rather than a true difference between a research and a non-research setting. For example, even in studies that we judged to be 'controlled', the absence of randomisation could mean that important differences between patients or the treatments they received will have been responsible for the outcomes, rather than the setting of their treatment. As others have shown, the correction for potential confounders in non-randomised studies does not guarantee that selection biases will be overcome [19]. Whereas in the 'poorly controlled' studies, it is even more likely that differences between the patients or their treatments led to at least some of the differences detected between the settings, as noted above.

Furthermore, we cannot rule out the possibility of publication or outcome reporting bias, in which either the availability of the studies we identified was influenced by their findings or the findings that the researchers chose to report were influenced by the magnitude of the effect [20,21]. For example, the majority of studies did not report on health outcomes and it is possible that these outcomes were measured, but then not reported because of their findings. It is not possible to examine this using, for example, research registers or accessible protocols because those sources are typically not available for studies of the type examined in this review.

Only three of the 13 included studies reported on the primary outcome for this review, health outcomes for patients in the different settings. Each of these compared the outcomes for patients treated in trial institutions versus non-trial institutions. The earliest of these three studies investigated acute myocardial infarction patients in Canada in the late 1980s and early 1990s, with a comparison between 40 research hospitals and 165 other hospitals. When patients in the two types of hospital were compared, after the exclusion of trial participants and using multivariable analyses to control for patient characteristics (age, sex, bypass grafting or coronary angioplasty), hospital mortality was similar between the trial and the non-trial hospitals [12]. A study from the early 2000s in Germany compared 80 hospitals that took part in clinical trials with 85 other hospitals, finding that deaths following a diagnosis of ovarian cancer were more likely in the non-trial hospitals after adjustment for baseline factors, including patients characteristics and hospital volume [13]. The third study, from the USA in the 2000s, compared 123 hospitals with high-trial participation, 226 hospitals with low trial participation and 145 hospitals with no-trial participation, finding that patients with non-ST-segment elevation acute coronary syndrome treated at hospitals that participated in trials had significantly lower mortality than patients treated at non-participating hospitals, after adjustment for confounding factors including total number of hospital beds, geographic region, revascularization capabilities, and teaching status of the hospitals and age, sex, race, insurance status, family history of coronary disease, medical history, and features of the initial clinical presentation for the patients [15].

The other studies examined a variety of outcomes around the care given to patients in the different settings, such as adherence to clinical guidelines or prescription of particular drugs. These process outcomes might be considered as surrogates for health outcomes, but without a thorough examination of the potential effects of the interventions that were reported it is not possible to conclude whether these were likely to be beneficial or harmful to the patients in the studies. Furthermore, if participation in a trial research is more likely to lead to a practitioner or a hospital using the findings of that trial, one should be cautious about the validity of using the results of a single trial, rather than the systematic review within which it should be placed, to influence practice [22].

### Implications for practice

The limited evidence currently available suggests that there might be beneficial effects for patients who receive non-trial treatment from practitioners or in institutions that take part in trials. The reasons for this difference are unclear, and the relatively small amount of research on this subject and the possible influence of confounding by patient characteristics mean that this conclusion should be viewed with caution and is not robust enough to influence practice.

### Implications for future studies

Among the challenges in interpreting the findings of the studies in this review is identifying the factors that might influence differences between patients treated by trial practitioners or in trial institutions compared to those treated elsewhere. Future research needs to minimise these differences. The optimal design might be to randomise patients to be treated in trial versus non-trial settings, but we realise that this is likely to be difficult or impossible to implement. We expect that most future research will continue to use cohort designs. Minimising the problems of confounding variables will be a key challenge and these variables include demographic characteristics of patients such as age and sex, and prognostic or clinical characteristics of relevance to the patient's underlying condition and likely outcomes. It will also be important to collect potential explanatory variables for any differences between trial and non-trial settings. Larger numbers of patients, practitioners and institutions should be included in future studies to have sufficient power to detect moderate differences. This is particularly important if the lack of a significant difference in a study is to be interpreted as there being truly no important difference, rather than a lack of power, in either direction.

## Conclusions

The available findings from existing research suggest that there might be a 'trial effect' of better outcomes, greater adherence to guidelines and more use of evidence by practitioners and institutions that take part in trials. However, the magnitude of this effect and the consequences for patient health are uncertain. The most robust conclusion may be that there is no apparent evidence that patients treated by practitioners or in institutions that take part in trials do worse than those treated elsewhere.

## Conflicts of interests

Both authors declare that they have no known conflict of interests, other than their general involvement in research including randomised trials and systematic reviews.

## Authors' contributions

MC checked each record retrieved by the searches and the references in the reports of the included studies to identify reports that might be eligible for the review. He checked full copies of some of the potentially eligible articles and agreed on the final set of studies to be included with KL. He checked the data extracted for each included study. He drafted the first version of this manuscript. KL checked the references in the reports of the included studies to identify reports that might be eligible for the review. She checked full copies of some of the potentially eligible articles and agreed on the final set of studies to be included with MC. She extracted data from each included study. She revised the initial version of this manuscript. Both authors approved the final manuscript.

## Supplementary Material

Additional file 1 **Search strategy for MEDLINE**. A description of the search run in OvidSP MEDLINE 1950 to Jan week 2 2009, on 23 January 2009. Click here for file
