# Supplementary material for: Effects on patients of their healthcare practitioner's or institution's participation in clinical trials: a systematic review
Source: Trials. 2011 Jan 20;12:16. doi: 10.1186/1745-6215-12-16 (PMC3036633; doi:10.1186/1745-6215-12-16)
Supplement: Additional file 1 — Search strategy for MEDLINE. A description of the search run in OvidSP MEDLINE 1950 to Jan week 2 2009, on 23 January 2009. [file 1745-6215-12-16-S1.DOC]

# Additional File 1

# Search strategy for MEDLINE

Search run in OvidSP MEDLINE 1950 to Jan week 2 2009, on 23 January 2009

|  | **Search terms** | **Results** |
| --- | --- | --- |
| 1 | randomized controlled trials as topic/ | 57160 |
| 2 | random allocation/ | 62415 |
| 3 | random$.tw. | 423048 |
| 4 | or/1-3 | 483626 |
| 5 | (outside adj3 (trial? or randomi?ed or rct? or program)).tw. | 583 |
| 6 | ((nonentry or non entry or nonenter$ or non enter$ or ‘not enter$’ or nonenrol$ or non enrol$ or ‘not enrol$’ or nonparticip$ or non particip$ or ‘not particip$’) adj3 (trial? or randomi?ed or rct?)).tw. | 130 |
| 7 | ((nonentry or non entry or nonenter$ or non enter$ or ‘not enter$’ or nonenrol$ or non enrol$ or ‘not enrol$’ or nonparticip$ or non particip$ or ‘not particip$’) adj3 patient?).tw. | 411 |
| 8 | ((nonrandom$ or non random$) adj3 (patient? or group? or case? or serie? or study or studies or trial?)).tw. | 7109 |
| 9 | (exclud$ adj3 randomi?ation).tw. | 98 |
| 10 | ((non participant? or nonparticipant?) adj3 group?).tw. | 78 |
| 11 | (patient? adj3 prefer$).tw. | 9143 |
| 12 | ((treatment or method?) adj3 prefer$).tw. | 9896 |
| 13 | (treatment adj3 (select$ or choose or chose or chosen or choice)).tw. | 35809 |
| 14 | ((own or patient? or by) adj choice).tw. | 1396 |
| 15 | ((standard or usual) adj practice).tw. | 1551 |
| 16 | ((refus$ or decline$) adj3 (participat$ or random$)).tw. | 1204 |
| 17 | ((non or ‘not ‘ or lack$ or withh$ or without or refus$ or decline$) adj3 consent).tw. | 2333 |
| 18 | (follow up adj3 register$).tw. | 275 |
| 19 | or/5-18 | 67518 |
| 20 | zelen.tw. | 51 |
| 21 | (4 and 19) or 20 | 11088 |
| 22 | clinical trial.pt. | 445354 |
| 23 | controlled clinical trial.pt. | 77815 |
| 24 | randomized controlled trial.pt. | 260879 |
| 25 | comparative study.pt. | 1403101 |
| 26 | cohort studies/ | 89449 |
| 27 | (preference adj (stud$ or trial?)).tw. | 209 |
| 28 | (cohort adj (stud$ or trial? or analysis)).tw. | 38787 |
| 29 | or/22-28 | 1839098 |
| 30 | humans/ | 10440235 |
| 31 | animals/ | 4287219 |
| 32 | 31 not (30 and 31) | 3220918 |
| 33 | editorial.pt. | 228019 |
| 34 | letter.pt. | 634687 |
| 35 | comment.pt. | 369010 |
| 36 | or/33-35 | 919239 |
| 37 | 29 not (32 or 36) | 1442064 |
| 38 | 21 and 37 | 6096 |
| 39 | 200703$.ed. | 62282 |
| 40 | 200704$.ed. | 52393 |
| 41 | 200705$.ed. | 58550 |
| 42 | 200706$.ed. | 58735 |
| 43 | 200707$.ed. | 55884 |
| 44 | 200708$.ed. | 57793 |
| 45 | 200709$.ed. | 59364 |
| 46 | 200710$.ed. | 59366 |
| 47 | 200711$.ed. | 51924 |
| 48 | 200712$.ed. | 75953 |
| 49 | 2008$.ed. | 719226 |
| 50 | 2009$.ed. | 23187 |
| 51 | or/39-50 | 1334657 |
| 52 | 38 and 51 | 866 |
| 53 | from 52 keep 1-866 | 866 |
| 54 | (outside adj3 (trial? or randomi?ed or rct? or program or programme or study or studies)).tw. | 1098 |
| 55 | ((nonentry or non entry or nonenter$ or non enter$ or ‘not enter$’ or nonenrol$ or non enrol$ or ‘not enrol$’ or nonparticip$ or non particip$ or ‘not particip$’ or ‘not tak? part’) adj3 (trial? or randomi?ed or rct? or program or programme or study or studies)).tw. | 512 |
| 56 | ((nonentry or non entry or nonenter$ or non enter$ or ‘not enter$’ or nonenrol$ or non enrol$ or ‘not enrol$’ or nonparticip$ or non particip$ or ‘not particip$’ or ‘not tak? part’) adj3 (doctor? or clinician? or physician? or surgeon? or GP or GPs or nurse? or nursing or practitioner? or specialist? or generalist? or therapist? or physiotherapist? or midwife or midwives or oncologist? or radiologist? or radiographer? or gyn?ecologist? or obstetrician? or neonatologist? or an?esthetist? or p?ediatrician? or dermatologist? or psychologist? or psychiatrist? or ophthalmologist? or optometrist? or geriatrician? or cardiologist? or orthodontist? or dentist? or healthcare worker? or health care worker? or healthcare professional? or health care professional?)).tw. | 175 |
| 57 | ((nonentry or non entry or nonenter$ or non enter$ or ‘not enter$’ or nonenrol$ or non enrol$ or ‘not enrol$’ or nonparticip$ or non particip$ or ‘not particip$’ or ‘not tak? part’) adj3 (site? or center or centre or centers or centres or hospital? or institution? or institutional or organi?ation? or unit or units or clinic or clinics or workplace? or surgery or surgeries or office? or department or departments or sector or sectors or setting or settings or practice or practices)).tw. | 199 |
| 58 | ((nonrandom$ or non random$ or ‘not random$’) adj3 (doctor? or clinician? or physician? or surgeon? or GP or GPs or nurse? or nursing or practitioner? or specialist? or generalist? or therapist? or physiotherapist? or midwife or midwives or oncologist? or radiologist? or radiographer? or gyn?ecologist? or obstetrician? or neonatologist? or an?esthetist? or p?ediatrician? or dermatologist? or psychologist? or psychiatrist? or ophthalmologist? or optometrist? or geriatrician? or cardiologist? or orthodontist? or dentist? or healthcare worker? or health care worker? or healthcare professional? or health care professional?)).tw. | 61 |
| 59 | ((nonrandom$ or non random$ or ‘not random$’) adj3 (site? or center or centre or centers or centres or hospital? or institution? or institutional or organi?ation? or unit or units or clinic or clinics or workplace? or surgery or surgeries or office? or department or departments or sector or sectors or setting or settings or practice or practices)).tw. | 1090 |
| 60 | ((recommend$ or influence$) adj5 (therap$ or care or management or treatment? or pathway? or policy or policies)).tw. | 67337 |
| 61 | ((increase$ or influence$ or accelerat$ or ‘more likely’ or ‘less likely’) adj5 (‘use’ or adopt$ or practice)).tw. | 37911 |
| 62 | ((translat$ or implement$ or adopt$) adj5 practice).tw. | 4990 |
| 63 | (impact adj5 (decision$ or policy or policies or care or management or pathway or treatment?)).tw. | 18174 |
| 64 | (improve$ adj5 (quality or care or therap$ or management or treatment? or pathway? or practice)).tw. | 143248 |
| 65 | ((affect$ or effect? or benefit$) adj3 particip$ adj6 (randomi?ed or rct? or trial? or study or studies or program or programme)).tw. | 576 |
| 66 | ((particip$ or ‘tak$ part’) adj6 (randomi?ed or rct? or trial? or study or studies or program or programme)).tw. | 76966 |
| 67 | or/54-59 | 3084 |
| 68 | or/60-64 | 260618 |
| 69 | 65 or (66 and 68) | 6022 |
| 70 | 67 or 69 | 9023 |
| 71 | 70 not 52 | 8954 |

Note: lines 1-53 represent an update of the search carried out for the review by Vist et al,[1] limited to records added to MEDLINE from 2007. Lines 54 onwards are not limited by date of entry to the database. Line 71 excludes results already downloaded and checked for relevance at line 53, to avoid duplication of effort.
